# Chondroitin Sulfate Proteoglycan 4 as a Marker for Aggressive Squamous Cell Carcinoma

**DOI:** 10.3390/cancers14225564

**Published:** 2022-11-13

**Authors:** Kathryn Chen, Joel Yong, Roland Zauner, Verena Wally, John Whitelock, Mila Sajinovic, Zlatko Kopecki, Kang Liang, Kieran Francis Scott, Albert Sleiman Mellick

**Affiliations:** 1 Ingham Institute for Applied Medical Research, Medicine, University of New South Wales, Liverpool, NSW 2170, Australia; 2 School of Medicine, Western Sydney University, Campbelltown, NSW 2560, Australia; 3 School of Chemical Engineering, University of New South Wales, Kensington, NSW 2033, Australia; 4 EB House Austria, Research Program for Molecular Therapy of Genodermatoses, Department of Dermatology & Allergology, University Hospital of the Paracelsus Medical University, 5020 Salzburg, Austria; 5 Graduate School of Biomedical Engineering, University of New South Wales, Kensington, NSW 2033, Australia; 6 Future Industries Institute, University of South Australia, Mawson Lakes, SA 5095, Australia

**Keywords:** chondroitin sulfate proteoglycan, cutaneous squamous cell carcinoma, head and neck squamous cell carcinoma, cancer biomarker, gene expression

## Abstract

**Simple Summary:**

Many solid tumours, such as those of the breast, colon, and prostate, have well established molecular markers of malignancy. However, in certain cancers, such as squamous cell carcinoma (SCC), few clinically useful biomarkers exist. Recently, several candidates that might be used for diagnosis in SCC have been proposed. The purpose of this review is to discuss chondroitin sulfate (CS) proteoglycan 4 (CSPG4) as a tumour biomarker and explain why its expression might be considered in clinical decision making for patients with SCCs, including those of the head and neck, and those arising from rare genetic disorders, such as epidermolysis bullosa (EB).

**Abstract:**

Chondroitin sulfate (CS) proteoglycan 4 (CSPG4) is a cell surface proteoglycan that is currently under investigation as a marker of cancer malignancy, and as a potential target of anticancer drug treatment. CSPG4 acts as a driver of tumourigenesis by regulating turnover of the extracellular matrix (ECM) to promote tumour cell invasion, migration as well as inflammation and angiogenesis. While CSPG4 has been widely studied in certain malignancies, such as melanoma, evidence is emerging from global gene expression studies, which suggests a role for CSPG4 in squamous cell carcinoma (SCC). While relatively treatable, lack of widely agreed upon diagnostic markers for SCCs is problematic, especially for clinicians managing certain patients, including those who are aged or infirm, as well as those with underlying conditions such as epidermolysis bullosa (EB), for which a delayed diagnosis is likely lethal. In this review, we have discussed the structure of CSPG4, and quantitatively analysed CSPG4 expression in the tissues and pathologies where it has been identified to determine the usefulness of CSPG4 expression as a diagnostic marker and therapeutic target in management of malignant SCC.

## 1. Introduction

Early diagnosis is key to survival of patients with cancer [1]. The modern medical oncologist has many tools to diagnose and assess a patient’s cancer, including non-invasive imaging methods, as well as histochemical examination of a tumour, or liquid biopsy [2,3,4]. Tumour biomarkers are cellular or biochemical signals produced by the tumour or patient, which can change over time or with treatment because of cell death or acquisition of drug resistance. Examples include the: (i) loss of expression of Her2 in breast tumours, following treatment with Herceptin^®^/trastuzumab [5]; or (ii) expression or suppression of PD1/PDL1, following immune therapy with Tecentriq^®^/atezolizumab [6,7]. In general terms, molecular or biochemical markers can be broadly described as factors, which are produced by neoplastic cells, or cells of the microenvironment (e.g., macrophages, eosinophils, neutrophils, etc.) in response to changes in clinical course [8]. They can be either ‘cell free’, or associated with a cell/tissue, and can be detected/measured in a biopsy or bodily fluids by serology or tissue immunophenotyping, and/or directly imaged (e.g., radiolabelled PSMA-11 in prostate cancer) [9,10,11]. Current tumour biomarkers provide information that: (i) directs diagnosis and prognosis; (ii) provides a way to monitor disease status; (iii) helps with assessing treatment efficacy; and (iv) aids in planning treatment [9,12,13,14,15]. Biomarkers can be assessed prognostically as an early indicator of spread, or longitudinally over the course of a treatment to determine response over time, and overall effectiveness of a therapy. They include proteins and peptides, carbohydrates and sugars, small molecular weight metabolic by-products, and or/nucleic acids (RNA/DNA). As many cancers are treatable if detected early, and as many cancer therapies are generally cytotoxic with a narrow therapeutic index, the identification and use of these ‘canaries in the cancer coal mine’ in recent years has proven to be a boon for the management of cancer. In fact, many cancers, such as breast cancer and prostate carcinoma have multiple well-defined effective early diagnostic markers used in clinical practice [5,16]. This contrasts with many SCCs where most currently available diagnostic aids remain broadly correlative. Measures such as tumour thickness, margin status and age, routinely used by clinicians, have all been found to be unreliable predictors of disease severity and metastatic spread [17,18,19]. The objective of this review has been to collate the information available from published functional studies, as well as published data from transcriptome analysis to assess CSPG4 as a marker of clinical course in malignant SCCs.

## 2. Need for Markers of Malignant SCC

SCCs are a diverse range of malignancies that may present in a wide variety of anatomical locations, and which have a common keratinocyte origin. Two of the most common types include cutaneous SCCs (cSCCs), which are often ultraviolet (UV) radiation induced [20], as well as head and neck squamous cell carcinomas (HNSCCs), which have both viral (HPV) and non-viral origins [21]. cSCC arises from epidermal keratinocytes and is the second most common type of non-melanoma skin cancer (NMSC), after basal cell carcinoma (BCC) [22,23]. Important non-modifiable risk factors for SCC, include fair skin, advanced age, family history and inherited disorders, such as xeroderma pigmentosum, albinism, epidermodysplasia verruciformis and epidermolysis bullosa (EB). The main environmental carcinogen implicated in general population cSCC is UV radiation exposure, with the shorter wavelength UVB variant shown to be more responsible for photocarcinogenesis than the longer wavelength UVA. Other risk factors include immunosuppression, ionising radiation, industrial carcinogens, photosensitising drugs, tobacco smoke, chronic inflammation, and HPV infection [12,13,14,22,23]. HNSCCs originate from upper gastrointestinal (GI) and respiratory tract squamous mucosal surfaces, including nasal cavities, paranasal sinuses, oral cavity, pharynx, and larynx [15,18]. Over 90% of head and neck cancers are HNSCCs [24]. Oral and pharyngeal SCCs have traditionally been associated with areca nut consumption, tobacco use, and/or alcohol abuse [25], however, in recent years there has been an increasing link between tumourigenesis and HPV infection, mainly HPV-16 [15]. HPV-associated HNSCCs have a distinct mutational, biological, and clinical signature. Consequently, HNSCC can be separated into HPV-negative and HPV-positive subtypes [15,19].

The mortality rate for different SCCs is variable. cSCCs have a relatively low death rate compared to other cancers (e.g., <1–3% for cSCC c.f. 10% for colorectal cancer) and a relatively low rate of metastases (3–7%) [25,26,27,28,29]; however, the overall number of patients diagnosed every year mean that the number of deaths from cSCCs remains very high [14,25,30]. Notably, NMSCs including cSCC, has now overtaken melanoma worldwide for number of deaths [25]. In addition, in certain patients, lack of effective treatments and rapid dissemination of disease, mean that the rate of death from SCC is significantly higher than the general population. This group includes older patients, those that are immunocompromised, those affected by albinism, and those patients with the recessive subtype of EB (RDEB) [31,32,33,34,35]. Immunosuppressed patients have a 2.32-fold greater risk of death due to SCC [35]. Those aged 80 and over have a mortality rate of 34 per 100,000 compared to 1.9 per 100,000 (age-standardised rate) (Australian Institute of Health & Welfare/AIHW 2016) [33]. In addition, in Africa, few people with albinism survive beyond 40 years of age due to metastatic cSCC [36], while the cumulative risk of death following cSCC diagnosis in RDEB patients has been measured at 67.8%, 80.2% and 90.1%, for ages 35, 45 and 55, respectively [37].

For HNSCCs the overall five-year progression free survival rate has been measured at 68.2% (from the AIHW, 2014) [38]. This rate varies with type and stage of disease at diagnosis. For example, HPV-associated oropharyngeal cancer (HPV-OC), which accounts for 25–30% of all HNSCCs, is associated with much higher rate of survival (~95%) [39]. In locally advanced disease (excluding HPV-OC), the five-year progression-free survival rate is approximately 40–50%. In patients with one metastasis the five -year survival is 35%, and patients with multiple metastases on diagnoses have a survival rate as low as 4% [19,40]. In this case, the relatively low survival rate in HNSCC can be attributed to the ineffectiveness of current clinical evaluation methods, as most patients diagnosed with late stage HNSCC show little evidence of a pre- or early malignant lesion [15]. In addition, the difficulty in detecting early malignancy-associated changes in HNSCC causes a high degree of uncertainty in staging, resulting in variability in disease management [19,40,41].

Taken together, the high prevalence in the community, and high mortality in certain cohorts, would suggest that there remains an unmet need for the identification and clinical development of effective diagnostic biomarkers in SCCs. For all patients the ability to identify residual disease and discriminate between responders and non-responders would greatly enhance treatment effectiveness. Recent work has revealed several potential molecular markers of aggressive SCCs, including nucleic acids [42,43,44], and proteins [45,46]. Notably, recent analysis of data generated by transcriptome analysis [e.g., The Cancer Genome Atlas (TCGA) Research Network, http://www.cancer.gov/tcga (accessed on 9 September 2022)] [47], as well as a growing body of work in related preclinical (cell culture & animal) models, strongly suggests that the specific and differential expression of chondroitin sulfate (CS) proteoglycan 4 (CSPG4) may be used a potential marker of clinical course in SCCs.

## 3. Structure and Function of CSPG4

CSPG4 is a cell surface proteoglycan with roles in normal growth and development, as well as disease [48,49,50,51,52,53,54]. The functions of CSPG4 largely centre around modulation of the extracellular matrix (ECM) and cell signalling, although roles in regulating the immune system have also been identified [55,56]. The CSPG4 proteoglycan was first identified in 1981, through serological and immunochemical testing of a melanoma-associated monoclonal antibody, as a highly immunogenic antigen expressed on human melanoma cell surfaces [57]. The human CSPG4 gene, located on chromosome 15:24q2, is composed of 10 exons, and the RNA sequence length is 8071 bp, encoding an open reading frame of 2322 amino acids (aa) [58]. CSPG4 is also known as the melanoma CS proteoglycan (MCSP), and/or the high molecular weight melanoma associated antigen (HMW-MAA) [59]. Studies have also identified the rat orthologue of CSPG4, termed nerve/glial antigen 2 (NG2) [60,61]. It is important to note that while much of the current knowledge about the function of human CSPG4 is derived from experiments using rodent NG2, any functional differences between the two orthologues (if any) remain unpublished [56,60].

CSPG4/NG2 has been implicated in a range of pathologies. Recently, a variation in the human CSPG4 gene has been shown to increase susceptibility to the Neurofibromatosis type 1-like phenotype, a phenomenon linked to the appearance of benign tumours and growths on nerves [62]. In spinal cord injuries, CSPG4/NG2 expressing cells accumulate at the site of injury, causing detrimental effects on axon regeneration in a process that has been shown to be mediated by CS side chains [63]. In fact, evidence seems to suggest that CSPG4/NG2 engages in significant crosstalk with the immune system via these CS side-chains. For example, injection of BALB/c mice with CS induces certain autoimmune diseases, such as rheumatoid arthritis, through aberrant recruitment and activation of CD4^+^ T cells [55]. A rare missense mutation found in the human CSPG4 gene has also been linked to reduced brain white matter integrity, and it has been suggested that in this instance misfunction of CSPG4 is a possible driver of familial schizophrenia [64].

### 3.1. CSPG4/NG2 Structure

**Figure 1:**
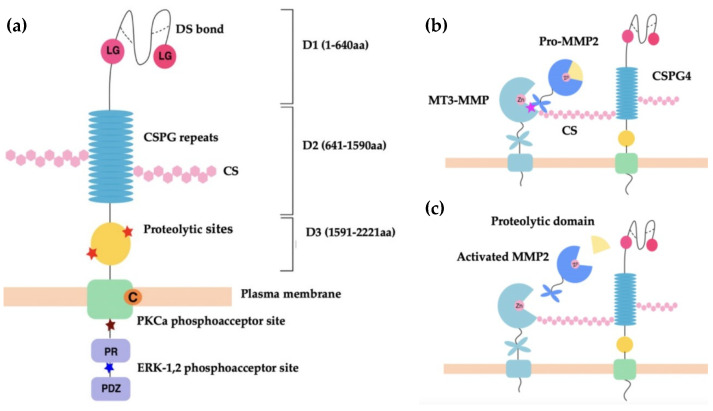
(**a**) Schematic representation of CSPG4 functional regions. Shown, three subdomains of the extracellular region, D1, D2 and D3 of CSPG4/NG2. The D1 structure is maintained by disulfide bonds (dotted lines). The laminin-type globular (LG binding region) in D1 mediates the binding of laminins. D2 contains 15 CSPG repeat sequences and multiple CS binding sites. D3 contains multiple proteolytic sites that are potentially MMP cleavage sites. Intracellularly, the PKC-α phosphoacceptor site is composed of threonine residue Thr^2252^ and represents a site of CSPG4 intracellular phosphorylation. The ERK1/2 phosphoacceptor site is also a threonine residue, Thr^2310^. The proline-rich region (PR) is responsible for additional poorly defined protein–protein interactions. The ‘PDZ domain’ binding motif binds the PDZ domain of proteins involved in architecture and scaffolding, such as synthenin (CSPG4), MUPP1 (CSPG4) and GRIP1 (NG2). The PDZ domain plays a role in protein scaffolding [59,65]. (**b**,**c**) Schematic representation of CSPG4 activation of pro-MMP2. The C-terminal hemopexin domain of pro-MMP-2 binds to the extracellular catalytic domain of MT3-MMP and the CS GAG of CSPG4 simultaneously to cause activation [56,66] (**b**), with the zinc binding site (Zn) of the catalytic domain acting to modulate selective inhibition of MMP activation [67]. Activation occurs when the proteolytic domain of pro-MMP-2 is cleaved to produce activated MMP-2, which is then released from the MT3-MMP and CSPG4 complex (**c**).

CSPG4 and its rat homologue NG2 are cell surface single-pass type I transmembrane (TM) proteoglycans [56]. CSPG4 and NG2 are both expressed as 250 kDa glycoproteins, which acquire CS glycosaminoglycans (GAGs) to produce 450 kDa proteoglycans [56,60]. Both GAG- and non-GAG-linked CSPG4 have been identified in cell membranes where it is clustered to lipid rafts [65]. The CSPG4/NG2 core protein consists of three structural regions: (i) a large extracellular domain (1–2221 aa) with three subdomains [D1 (1–640 aa), D2 (641–1590 aa), D3 (1591–2221 aa)]; (ii) a TM region; and (iii) a cytoplasmic C terminal domain (CTD) (Figure 1a) [56].

The membrane distal D1 subdomain serves as an N-terminal globular domain with two laminin G-type regions and multiple disulfide bonds that maintain tertiary structure [56]. D1 contains laminin G modules, which are implicated in basement membrane assembly and organisation, and in association with laminins play an important role in regulating cell-matrix adhesion [68,69]. In addition, D1 contains a matrix metalloproteinase (MMP)-14 cleavage site, which permits release of CSPG4/NG2 into the environment [68]. The D2 domain is composed of 15 repeat amino acid sequences, some of which are binding sites for CS, or act as potential glycosylation sites [60]. The D2 subdomain is proposed to interact with collagens, growth factors, metalloproteinases, as well as integrins via these CS GAG elements [56,66,67,68,69,70,71,72]. The D3 subdomain contains multiple putative proteolytic sites and carbohydrate decorations that potentially interact with lectins and integrins [73,74].

The TM domain (2222–2246 aa) contains a cysteine residue at position 2230 that may have a function in controlling CSPG4/NG2 membrane localisation. The CTD contains 2 threonine residues that act as phosphoacceptor sites for protein kinase C-α (PKC-α), and the extracellular signal-regulated kinase (ERK1/2). Differential phosphorylation of these threonine sites occurs depending on the cellular events that CSPG4 is involved in [59,75,76]. This domain also contains a proline-rich region and a four residue PDZ domain-binding motif at the C terminus [56,60]. The CTD is linked to the intracellular actin cytoskeleton via PDZ-type adaptor proteins, such as MUPP1 and synthenin-1, as well as through association with ezrin and cofilin-1 [77,78]. As a result of PKCα/ERK1/2-regulated threonine phosphorylation and intracellular cytoskeletal interactions, the CTD of CSPG4/NG2 is highly involved in receptor tyrosine kinase signalling through the mitogen-activated protein kinase (MAPK), and integrin/focal adhesion kinase (FAK) signalling [79,80,81].

### 3.2. CSPG4 Function in Malignancy

CSPG4/NG2 regulates various cellular processes related to tumourigenesis, including cytoskeletal reorganisation, adhesion, motility, migration, metastasis, the epithelial-to-mesenchymal transition (EMT), growth, survival, and chemoresistance [54,56,59,76,82,83]. In cancer cells, NG2/CSPG4 is expressed at the advancing membrane front, specifically at the leading edge of filopodia forming microspikes [60,84]. Moreover, several studies have linked CSPG4 expression to the development of certain cellular traits necessary for tumour progression. For example, increased CSPG4 levels has been linked to superior engraftment ability and enhanced local growth of sarcoma cells in culture settings [85]. In genetically modified mice, enhanced expression of NG2 has also been shown to cause increased rates of metastasis in melanoma [86]. How CSPG4 regulates the ECM and other cellular processes to effect malignancy associated changes is the subject of the next part of this review.

#### 3.2.1. Regulation of Extracellular Proteases

There is an increasing body of evidence, which suggests that CSPG4 affects changes to the ECM by mediating activation of extracellular proteases, including MMPs [56,67,73]. MMPs are zinc-dependent multifunctional multidomain proteins that proteolyse components of the ECM for: (i) tissue turnover (laminin, collagen, fibronectin, gelatin, etc.), (ii) releasing bioactive products, (iii) participating in (mediate) membrane shedding, as well as (iv) regulating chemokine processing and proenzyme activation [87]. MMPs therefore play key roles in both maintenance of tissue homeostasis and tissue regeneration during wound healing [88]. The best described relationship between MMPs and CSPG4 is that between CSPG4, the membrane-type (MT) 3-MMP, and gelatinase A (MMP-2) (Figure 1b). In this instance, CSPG4 facilitates the MT3-MMP mediated conversion of pro-MMP-2 into active gelatinase A [66]. Notably, unlike other MMPs, MMP-2 seems to be dependent on this form of activation at the cell membrane, which it utilises to facilitate the localised breakdown of ECM at the leading edge of cell movement. In cancer, MMP-2 activity facilitates localised tumour cell invasion into neighbouring tissue and is involved in vascular remodelling, as well as neoangiogenesis [66,67]. Since MMP-2 is a ubiquitous protease, it is also involved in other pathologies, with links to cell migration and tissue turnover, such as inflammation, and atherosclerotic plaque rupture [89,90]. In the cases described above, CSPG4 plays an indirect role in regulating cell function and growth factor activation by mediating MMP activity. However, CSPG4 may also play a more direct role in disease and development by acting as a co-receptor for ligand binding and receptor activation.

#### 3.2.2. CSPG4 as a Co-Receptor

**Figure 2:**
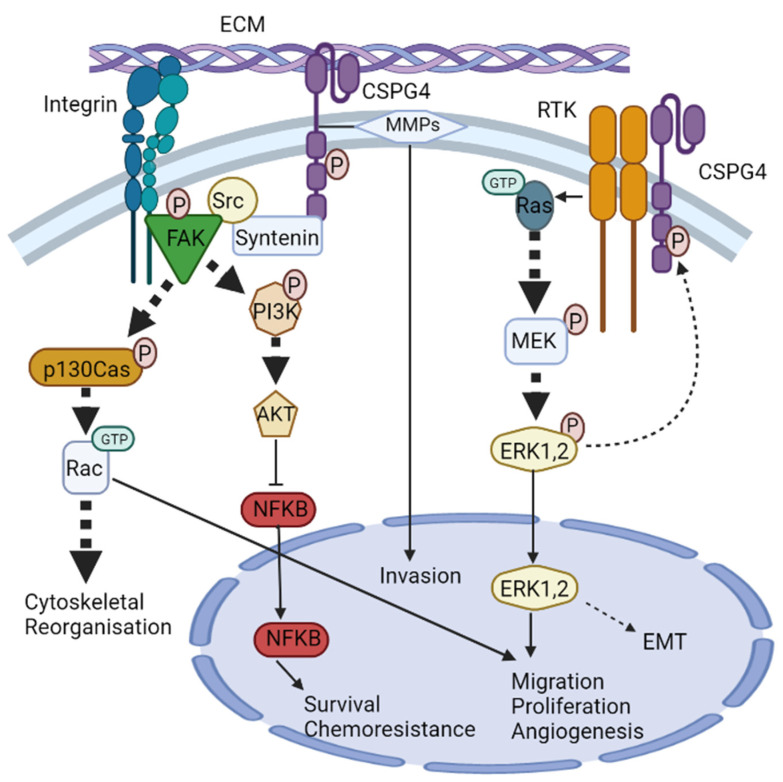
CSPG4 signalling pathways. CSPG4 activates two major signalling cascades via its cytoplasmic domain: (i) the FAK integrin signalling pathway and (ii) the MAPK/ERK1/2 pathway. (i) The scaffold protein syntenin physically interacts with Src, and at the same time, integrin engagement recruits and stimulates the autophosphorylation of FAK. Syntenin then promotes FAK/Src complex formation to initiate phosphorylation of p130cas and PI3K. p130Cas phosphorylation initiates activation of the small Rho GTPase Rac, leading to cytoskeletal reorganisation. PI3K phosphorylation activates AKT which regulates transcriptional activity of NF-κB, leading to survival chemoresistance. (ii) CSPG4 activates MAPK/ERK1/2 signalling through RTK-dependent and independent mechanisms. Activation of CSPG4 causes activation of small GTPase Ras, which stimulates MEK phosphorylation and then ERK1/2 phosphorylation. ERK1/2 then unregulated MITF and c-Met leading to enhanced EMT. ERK1/2 also has other targets, and along with Rac, promotes migration, proliferation, and angiogenesis.

CSPG4/NG2 is devoid of innate catalytic activity [56,91], but the core protein serves as a co-receptor that has been shown to bind the basic fibroblast growth factor (bFGF/FGF2), as well as platelet-derived growth factor (PDGF)-AA [92,93]. CSPG4-bound growth factors have been shown to be resistant to degradation; hence CSPG4/NG2, much like many other proteoglycans, also functions as an extracellular reservoir for growth factors and facilitates growth factor binding to signal transducing receptors [81,94,95]. FGF2 is implicated in cell proliferation, migration, differentiation, and angiogenesis [95]. PDGF-AA binds to a dimer of two PDGF-α receptor tyrosine kinases and enacts its paracrine function of enhancing the EMT, growth, angiogenesis, invasion, and metastasis [96]. The effect of these growth factors is mediated through tyrosine kinase signalling (e.g., Ras-MEK-ERK1/2 pathway) [56,91] (Figure 2). The ability of CSPG4 to enhance ERK1/2 signalling is also amplified by the genomic landscape of patients with melanoma, as approximately 60% of human melanomas have been shown to express the constitutively active BRAFV600E mutant, which drives constitutive ERK1/2 phosphorylation and activation, likely by impacting growth factor-induced activation of Ras tyrosine kinases (RTKs). Several studies have shown that BRAFV600E cannot maintain maximal ERK1/2 activation without the expression of CSPG4 [76,97].

#### 3.2.3. CSPG4 & Integrin Signalling

In addition to acting as a co-receptor for growth factor and cytokine signalling, CSPG4 has also been shown to interact with key TM mediators of external signals, notably the integrins. Integrins and cell-surface proteoglycans are implicated in cell–cell and cell-ECM interactions with critical roles during wound healing and tumour development. Integrins are a family of 24 different proteins that form heterodimers and are the principal receptors for binding to ECM. Their primary function is to link the actin cytoskeleton to components of the ECM [98]. Unsurprisingly, CSPG4 has also become implicated in integrin activation. For instance, integrin α4β1 can become super-activated in response to ECM ligands (such as fibronectin) when activated in the presence of CSPG4 [56,99,100]. This is because CSPG4 not only acts as a co-receptor for integrin but also enhances α4β1 function by stimulating downstream adhesion-related intracellular signalling pathways involving cytoplasmic tyrosine kinases, such as the focal adhesion kinase (FAK), and ERK1/2. The result of this integrin ‘super activation’ is independently enhanced levels of FAK and ERK1/2, compared to integrin that is activated in the absence of CSPG4 [80,99].

Another pathway by which CSPG4 enhances integrin activity is through intracellular signalling involving Cdc42 (a member of the Rho family of GTPases), the activated-Cdc42-associated kinase-1 (Ack1) and p130cas [83], all of which have been shown to be critical in transmitting mechanical forces and managing actin dynamics governing SCC development and progression [100,101]. Clustering of CSPG4 has been shown to activate Cdc42 to a GTP-conjugated state. A complex of activated Cdc42 and Ack1 is then recruited, which in turn phosphorylates p130cas. p130cas and enhances α4β1 integrin action to cause actin cytoskeletal reorganisation via microspike formation [102]. Cdc42 has also been shown to interact with the phosphatidylinositol-3-kinase (PI3K), and potentially FAK, to further regulate integrin β1 adhesive signals and promote tumour survival [56,83]. Notably, enhanced α4β1 expression is directly linked to growth, invasion, migration, and metastasis in different cancers, including melanoma [103]. CSPG4 has also been shown to mediate cell spreading in human astrocytoma cells via p130cas and another Rho family GTPase, Rac [104]. Furthermore, at least one study has shown that CSPG4-mediated activation of integrin enhances resistance to chemotherapy by sustained activation of the AKT pathway via PI3K [81]. The role of CSPG4 in modulating the cytoskeleton makes it an ideal target of modern anticancer research, given the current interest in proteins involved in regulating mechanical forces and actin dynamics [101].

#### 3.2.4. CSPG4 as a Receptor for Structural Components of the ECM

In addition to working with membrane-bound factors to transmit signals from the ECM, CSPG4 has been shown to mediate cell function and subsequent pathology during tumour development via direct binding of ECM components. For instance, the extracellular domain of CSPG4 acts as a receptor for several structural components of the ECM, directly binding collagen types II, V and VI [71]. However, the relationship between CSPG4 and other members of the ECM, such as laminin, tenascin, and fibronectin remain less clear, and is probably more indirect. In addition to directly linking structural components of the ECM, studies have shown that CSPG4 strengthens existing functional linkages between ECM components, and growth factor receptor/integrin mediated pro-malignant signalling pathways [67,78,81,86]. CSPG4/NG2 has been reported to bind to perlecan, a heparan sulfate (HS) proteoglycan present in the ECM, which in concert with α2β1 integrin acts to support cell adhesion [105]. Additionally, upregulation of collagen VI in soft tissue sarcoma has been shown to enhance CSPG4 mediated malignancy [79]. CSPG4 also seems to enhance malignant cell interactions with the stromal ECM by activating pro-survival signalling cascades, thereby enhancing anti-cancer drug resistance [56,77,79,81,106].

Laminins are essential components of basement membranes and have critical functions in transmitting extracellular communications via membrane proteins to cytoplasmic effectors. Many functions such as basement membrane assembly and organisation, as well as cell-matrix adhesion are regulated by laminins [68,69]. The D1 subdomain consists of a globular N-terminus containing eight cysteines and four serine/glycine pairs as well as two laminin G-type motifs. It is through these laminin g domains that CSPG4 is thought to mediate (and sometimes compete with) cellular receptors (integrins, α-dystroglycan), sulfated carbohydrates, and other extracellular ligands, for laminin binding [68]. CSPG4 may also enhance integrin signalling binding by sequestering integrin to membrane specific locations to facilitate movement at the leading edge of cell migration [75,76,107].

Tenascins are a family of large ECM glycoproteins, characterized by a six-armed quaternary structure and a modular construction [108]. As a prominent member tenascin C (TNC) is composed of four subunits: a cysteine-rich amino terminal domain, a sequence of epidermal growth factor (EGF)-like repeats, fibronectin type III repeats, and a carboxy-terminal domain homologous to fibrinogen. Tenascin is upregulated with CSPG4 in wound healing and development, and it has been proposed that dysregulated expression of both CSPG4 and tenascin is a key feature of many pathologies, including cancer [109,110]. Notably, TNC binds soluble fibronectin and blocking TNC binding to CSPG4 via Syndecan-4 addition has been shown to reduce tumour cell adhesion and migration [111].

Fibronectin is an abundant ECM glycoprotein that is involved in wound healing and repair and is also an important component of blood clots. Fibronectin interacts with other ECM members including collagen, and GAGs [112]. While fibronectin has been shown to interact with CSPG4 [100], where it plays a role in enhancing the stability of complexes at the leading edge of cell migration (possibly via TNC-mediated binding) [113]. In this case, directional migration is probably dependent on intrinsic factors associated with the cell, as well as the polarised morphology of a migrating cell [114]. Notably, polarised morphology is closely linked to factors associated with cellular differentiation, including those linked to the EMT [115].

#### 3.2.5. c-Met, CSPG4 and the EMT

Pathological EMT involves many processes that are similar to developmental EMT (a process of cellular differentiation which occurs during embryogenesis), and is observed during tissue regeneration, organ fibrosis, and wound healing. Each of these processes is associated with specific molecular changes, which include but are not restricted to E-cadherin loss (loss of epithelial phenotype), acquisition of mesenchymal cadherins (N-cadherin & CDH11), and increased expression of the cytoskeletal filament, vimentin, as well as ECM constituents, such as fibronectin. Altered EMT programs have been found to be a necessary part of metastatic expansion, enabling tumour cells to acquire a morphology that is more suited for extracellular migration and settlement at distant sites [56,115,116].

In melanoma, an analogous process to the EMT has been shown to occur. In this case, cell migration and anchorage-dependent growth requires the expression and activation of the mesenchymal epithelial transition tyrosine kinase/hepatocyte growth factor receptor (c-Met). Inhibiting c-Met expression has been shown to limit the growth, invasiveness, and motility of melanoma cells [60,82]. Notably, human melanoma cells expressing CSPG4 also exhibit constitutive activation of ERK1/2, a process that requires the presence of intact CSPG4 core protein and cytoplasmic domain. ERK1/2 activation in turn leads to upregulation of the microphthalmia-associated transcription factor (MITF), causing enhanced expression of c-Met. In human melanoma, inhibition of MEK1, an ERK1/2 activator, has been shown to halt expression of both MITF, and c-Met. In addition, treating cells with siRNA against MITF inhibits c-Met expression, supporting the link between CSPG4 and cellular differentiation associated with the acquisition of a malignant phenotype through stimulation of c-Met expression via activation of ERK1/2/MITF/c-Met [82]. However, the switch in gene expression patterning from an epithelial to a mesenchymal one is much more complex than simply the activation of c-Met and involves the activation several transcriptional networks mediated by: (i) transcription factors, such as SNAI1, SNAI2, ZEB1, ZEB2, Twist, and E12/E47, (ii) non-coding RNAs, (iii) epigenetic modifications, (iv) alternative splicing, (v) post-translational regulation, and (vi) subcellular localisation [115,116]. While the interaction between CSPG4 function and c-Met is well described the mechanism (if any) by which CSPG4 might affect these other EMT-linked pathways should be the subject for future study. In addition to the EMT, CSPG has also been shown to regulate other processes of cellular differentiation related to cancer malignancy, notably angiogenesis.

#### 3.2.6. CSPG4 & Angiogenesis

CSPG4/NG2 has been shown to promote angiogenesis, in two ways: (i) by directly controlling behaviour of neovasculature; and (ii) by indirectly modulating angiocrine factors [54,117,118,119,120,121,122]. Angiocrine factors that are known to be influenced by NG2/CSPG4 include FGF-2 and PDGF [92,93,123]. CSPG4 is expressed on pericytes which are branched cells within the basement membrane of capillaries and venous microvasculature that are known to promote angiogenesis and neovascularisation [57,124,125,126]. CSPG4/NG2 does not have to be localised to cell membranes to produce an effect on angiogenesis, as soluble CSPG4/NG2 released from tumour cells has also been shown play an important role in development and pathogenesis. In addition, by modulating angiocrine factors, CSPG4 also enhances pro-angiogenic interactions between pericytes, endothelium and malignant cells [118,124,125,126]. One of the mechanisms by which this occurs is through galectin 3 and α3β1 integrins, which act to enhance endothelial cell migration [74,118]. Moreover, in melanoma CSPG4 modifies the NF-κB pathway to promote tumour angiogenesis [56,127], and CS groups present on CSPG4 have been shown to act as ligands for P-selectin, a cell adhesion molecule on the surface of activated endothelial cells [128]. The result of enhanced angiogenesis is the supply of nutrients and oxygen to the tumour, which enhances growth, and enables metastasis to occur haematologically.

## 4. CSPG4 Expression in Disease and Development

Several studies have identified CSPG4 as a possible marker of malignancy in a range of tumours, including pancreatic cancer, leukemia, melanoma, and glioma [129,130,131]. In melanoma, elevated soluble CSPG4 has been identified in the blood, although there remains debate about any relationship between plasma CSPG4 levels and patient prognosis [132,133]. Elevated expression of CSPG4 has also been identified in SCCs, compared with keratinocytes [43,49,134]. However, while CSPG4′s potential as a drug target and marker has been explored in other malignancies, such as melanoma, there has been less focus on CSPG4 as a putative biomarker and therapeutic target in aggressive SCCs.

### 4.1. An Overview of Methodological Approach

In the remaining part of this review, we have used a combination of literature searching, and reanalysis of published data to provide an overall snapshot of CSPGP4 expression in cancer with a focus on SCCs. For accessing information from TCGA database we used the GEPIA web browser tool [47]. To compare expression between cancer and control (noncancer) tissues one-way ANOVA was used (α = 0.05). Transcriptome data from recent public data on RDEB SCCs and cell lines was also reanalysed [43,44], using nonparametric Mann–Whitney U analysis (α = 0.05), to compare medians (α = 0.05). Survival analysis was conducted using Kaplan–Meier survival (α = 0.05), and comparison of tumour stage using one-way ANOVA (α = 0.05). Ontology analysis was conducted using OMIM^®^ designations [135]. Pearson correlation (α = 0.05) was used to identify genes that coregulated with CSPG4 in HNSCCs as well as RDEB SCCs (α = 0.05). The correlation coefficient (r) is provided where available. Unless otherwise stated the online GEPIA web-based tool or GraphPad Prism^®^ was used for reanalysis and presentation of data.

### 4.2. Tissue Distribution of CSPG4

CSPG4/NG2 is expressed in normal tissues throughout development, with all reports pointing to an undeniable ubiquitous role for this glycoprotein in embryonic organogenesis and in adult tissue homeostasis (Table 1). However, because CSPG4 function is complex, and multivariate, it is not yet fully understood. Interestingly, protein levels do not always correspond to degree of expression. Such is the case for brain tissue in which RNA levels are relatively low, compared to protein levels which are relatively high. The regulation of CSPG4 activity has been shown to be affected by immune system activity, epigenetics, transcription factor regulation and microRNA action [49,60,134].

In the embryo, CSPG4/NG2 expression is enhanced in mouse mesenchymal chondroblasts, particularly during the stage of growth where the chondroblasts differentiate into chondrocytes. NG2 downregulation occurs after differentiation is complete. A similar pattern occurs in the osteoblast to osteocyte transition, suggesting that NG2 has a role in endochondral and intramembranous ossification [136]. In vasculogenesis, NG2 is expressed by the mural component of neovascular structures including in embryonic cardiomyocytes, smooth muscle cells in microvasculature, and in pericytes in microvasculature. NG2 is absent in mature endothelium, suggesting a role in the formation of new vascular structures (angiogenesis) [52,53,54,119].

In the adult, CSPG4 is expressed by several multipotent progenitor cell populations, which originate within the interfollicular epidermis. In this case, CSPG4 is believed to play a role in localisation and maintenance of these cells within their niche [139,140]. Notably, loss of CSPG4 by epidermal stem cells has been linked to skin aging [141]. Within the CNS, CSPG4/NG2 expression is a hallmark of oligodendrocyte, astrocyte, and neuronal precursors, where it likely plays a role in their differentiation, as well as establishment of the neuronal network [123,142]. In the adult skin, CSPG4 expression appears to be restricted to stem cells, with large scale protein and gene expression projects showing CSPG4 expression in the healthy skin to be relatively low [47,49]. Along with being expressed in embryonic mural components of neovasculature, NG2 has also been found in adult mouse pericytes suggesting a role in both vasculogenesis and angiogenesis [52,54,137]. Notably, knockout of NG2 in mice results in a severe lack of pericyte recruitment to new blood vessels in corneal and retinal models, as well as an overall reduction in neovascularisation [117,118]; effects that are possibly due to a lack of pericyte-dependent β1 integrin activation on endothelial cells [144]. Taken as a whole, an analysis of the distribution of CSPG4 shows that it is expressed in a very wide range of both foetal and adult cells, but the mechanism of action of CSPG4 in normal development and tissue homeostasis remains poorly defined.

### 4.3. CSPG4 Expression in Malignancy

**Figure 3:**
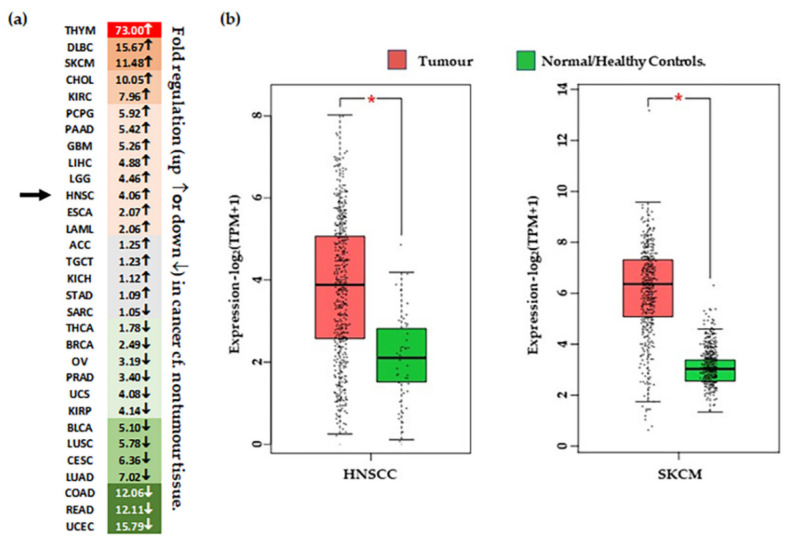
Differential expression of CSPG4 in tumour tissue. (**a**) Heat map of ‘fold’ difference (up ↑, or down ↓) between control and tumour tissue across different malignancies. See abbreviations below. Green represents down regulated gene expression compared to normal (noncancer) tissue and red represents increased expression compared with normal (noncancer tissues). Grey indicates a difference that is minor (<1.5-fold up or down). Arrows indicated fold change in HNSCC. (**b**) CSPG4 expression in HNSCC [Left, *n* (tumour tissue) = 519 & *n* (/healthy tissue) = 44], and melanoma [Right, *n* (tumour tissue) = 461 & *n* (normal/healthy tissue) = 558]. Shown, significantly higher expression of CSPG4 in HNSCCs and melanoma compared with healthy tissues (by one-way ANOVA, α = 0.01, * *p* < 0.01). Data (transcripts per million/TPM) is transformed [log_2_ (TPM+1)], and represented as box plots, showing median values and quartiles. Figure derived from analysis of publicly available TCGA Data, accessed through the GEPIA portal [47]. ACC–Adrenocortical carcinoma; BLCA–Bladder Urothelial Carcinoma; BRCA–reast invasive carcinoma; CESC–Cervical squamous cell carcinoma & endocervical adenocarcinoma; CHOL–Cholangio carcinoma; COAD-Colon adenocarcinoma; DLBC–Lymphoid Neoplasm Diffuse Large B-cell Lymphoma; ESCA–Esophageal carcinoma; GBM–Glioblastoma multiforme; HNSC–Head & Neck SCC; KICH–Kidney Chromophobe; KIRC–Kidney renal clear cell carcinoma; KIRP–Kidney renal papillary cell carcinoma; LAML–Acute Myeloid Leukemia; LGG–Brain Lower Grade Glioma; LIHC–Liver hepatocellular carcinoma; LUAD–Lung adenocarcinoma; LUSC–Lung squamous cell carcinoma; MESO–Mesothelioma; OV–Ovarian serous cystadenocarcinoma; PAAD–Pancreatic adenocarcinoma; PCPG–Pheochromocytoma & Paraganglioma; PRAD–Prostate adenocarcinoma; READ–Rectum adenocarcinoma; SARC–Sarcoma; SKCM–Skin Cutaneous Melanoma; STAD–Stomach adenocarcinoma; TGCT–Testicular Germ Cell Tumours; THCA–Thyroid carcinoma; THYM–Thymoma; UCEC–Uterine Corpus Endometrial Carcinoma; UCS–Uterine Carcinosarcoma; UVM–Uveal Melanoma.

To assess the role of CSPG4 in malignancy, we next reviewed primary papers, and data from transcriptome analysis available through the: (i) TCGA, accessed using the GEPIA (Gene Expression Profiling Interactive Analysis) analysis tool [47], and (ii) Genotype-Tissue Expression (GTEx) data, available through the online portal [51] (Table 2). Data was also collected from recent transcriptome analysis performed on cSCCs from patients with RDEB [43,145]. Following analysis of TCGA data, transcripts for CSPG4 were found to be significantly higher in several cancers compared to normal tissues, most notably HNSCC, and glioblastoma (Figure 3). Interestingly, CSPG4 is also highly expressed at multiple stages of melanoma, even in pre-malignant naevi [47,56]. In line with TCGA and GTEx data, Warta et al. (2014) [134] found that mean CSPG4 mRNA expression in HNSCC cells was 26.6 times higher than non-tumour cells. Additionally, Warta et al.’s analysis of three public HNSCC gene expression datasets found that CSPG4 expression was substantially higher in malignant lesions compared to normal tissue.

**Figure 4:**
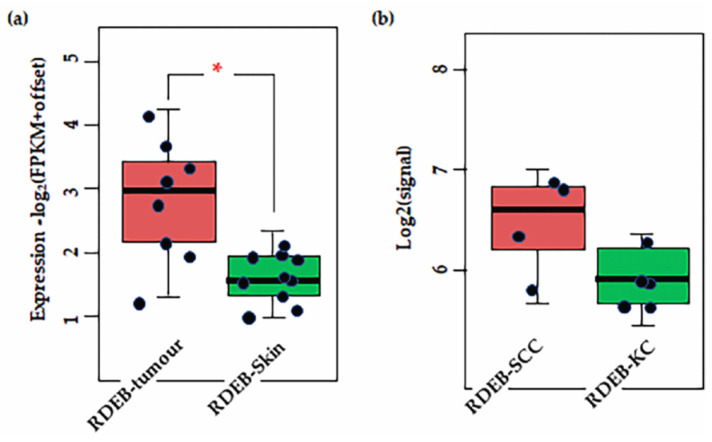
Results of transcriptome analysis of (**a**) RDEB tissues (**b**) and cell lines. Shown significant difference is CSPG4 expression between RDEB tumour tissue and non-tumour skin (* *p* = 0.0031, by Mann–Whitney U, α = 0.05). Notably, there is a slight (not significant, by Mann–Whitney *p* = 0.48, α = 0.05) difference in expression between primary RDEB keratinocytes and RDEB-SCCs, enriched and maintained in culture. Although the numbers in are small (*n* = 5 and *n* = 4, respectively). Data is represented as fragments/kb of exon (FPKM)/1 × 10^6^ mapped fragments. To prevent log (0) offset established as 0.1. Figure derived from re-analysis of publicly available published data: GSE130925 [43] and GSE130925 [145].

There is a lack of data for cSCC and CSPG4 from the TCGA, but primary data exists from Wilson et al. (1981) that shows higher levels of CSPG4 expression in BCCs and SCCs, compared with normal skin [57]. In addition, recent transcriptome re-analysis of cell lines and tissues from the rare RDEB population, where SCC diagnosis is almost always lethal, showed that CSPG4 expression is significantly higher in RDEB cSCCs than in RDEB skin [43,145] (*p* = 0.0031, by *ANOVA*, α = 0.05) (Figure 4a). Although the number of tissues in this study was small, this finding does correlate with the higher level of CSPG4 expression in SCCs in the general population. Notably, a slight increase in CSPG4 expression was also observed in primary RDEB-SCCs, compared with RDEB keratinocytes in culture (Figure 4b). Although this was not significant (*p* > 0.05) the number of cell lines investigated in this study was small, and a larger sample should be investigated to determine whether this apparent drop in CSPG4 expression, is a result of the cells responding to the lack of a normal ECM, or an artifact of 2D cell culture.

### 4.4. CSPG4 Expression, Patient Survival & Stage

**Figure 5:**
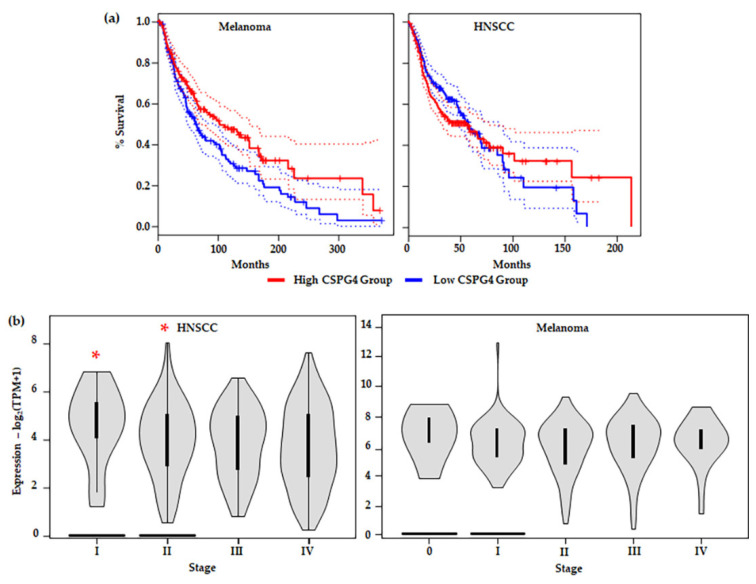
(**a**) Survival analysis comparing tumours with CSPG4_high_ (top 50%) and CSPG4_low_ (lower 50%) expressing groups in: (***Left***) melanoma (CSPG4_high_, *n* = 229 & CSPG4_low_, *n* = 229); and (***Right***) HNSCC (CSPG4_high_, *n* = 229 & CSPG4_low_, *n* = 229). Shown, significant overall survival (OS) in CSPG4_high_, compared with CSPG4_low_ s in melanoma {by Kaplan–Meier, α = 005, Logrank *p* = 0.0082, Hazards ratio/HR (CSPG4_high_) = 0.7, *p*-value for hazards ratio [*p*(HR)] = 0.0086}. Additionally, shown, no significant OS in CSPG4_high_, compared with CSPG4_low_ in HNSCC [α = 005, Logrank *p* = 0.35, HR (CSPG4_high_) = 1.1, *p*(HR) = 0.35] [47]. (**b**) Stage plots showing CSPG4 expression in different clinical stages, by TNM classification: (***Left***) HNSCC [F_value_ = 3.06, Pr (>F) = 0.0277, by *ANOVA*] and (***Right***) melanoma [F_value_ = 0.847, Pr (>F) = 0.496, by *ANOVA*]. Note the significant difference in CSPG4 expression between Stage I & II (non-metastatic tumours), compared with Stage III & IV (metastatic tumours) (* *p* < 0.05, α = 0.05). (**a**,**b**) derived from analysis of publicly available ACGT Data, accessed through the GEPIA portal [47].

In many of the studies outlined above, including in melanoma, high expression of CSPG4 in tumour issues and cell lines, compared to non-tumour tissue or healthy cells, has been associated with an aggressive tumour phenotype. Interestingly, examination of the TGCA data set revealed that higher CSPG4 expression in patient tissues is linked to significantly increased overall patient survival in melanoma (by Kaplan–Meier, * *p* = 0.0082) (Figure 5a) [47]. This result is surprising and may be due to the relationship between CSPG4 and tissue remodelling associated with a high inflammatory response and strong immune system in some patients [153,154]. In some cancers it has been shown that for some patients undergoing immune therapy, higher levels of CD8^+^ T cells in peripheral blood correlate with an increased localised immune response, an increased positive response to treatment, and improved survival [106,155]. However, further investigation of the link between CSPG4 expression and patient response to (immune) therapy in melanoma remains largely unexplored. Notably, no significant improvement in survival was observed in HNSCCs expressing a higher level (top 50%) of CSPG4, compared with those expressing lower CSPG4 (Figure 5a). In addition, in the first 50 months the high expressing group seems to have a lower survival, although this is not maintained over the course of the study. This early divergence in survival between melanoma and HNSCC shows promise, however, more work needs to be done to examine if this represents a real difference in pathophysiology of the two neoplasia.

When different tumour stages (TNM classification) [41] were compared, a significant difference in CSPG4 expression was observed between early (Stage I & II) and late stage (Stage III & IV) HNSCCs; with lower expression in local and distal metastatic stages (III & IV) (by one-way ANOVA, α = 0.05, *p* = 0.0277) (Figure 5b). In this instance, tumour mRNA for CSPG4 inversely correlated with metastatic tumour spread. This result appears to contradict previously reported cell culture and mouse models of cancer [60,78,79,128,130,134,156] where expression of CSPG4 is linked to tumour aggressiveness. In Stage I and II tumours, high CSPG4 expression may reflect an initial inflammatory response, and recruitment of bone marrow-derived cells, as well as tumour neovasculature, to the developing tumour. Early tumour development is characterised by a hypoxic adenoma, followed by an inflammatory response, and rapid neovascularisation leading (ultimately) to a very different tumour microenvironment, which is associated with a mature vasculature, reduced hypoxia, and stable microenvironment. This is sometimes referred to as the angiogenic switch [157,158,159]. The drop in CSPG4 expression in late tumours which have metastasised (undergone an angiogenic switch) may reflect this change in tumour architecture, as well as an overall reduction in widespread tissue remodelling. In fact, this hypothesis is supported by a recent report by Minaei et al. (2022), which identified lower CSPG4 expression in primary SCCs that had metastasised [42]. No similar significant reduction in CSPG4 expression between early non-metastatic (Stage 0-II) and late metastatic (Stage III & IV) tumours was observed in melanoma (Figure 5b). A divergence from that observed in HNSCC, which may be explained by the differing responses to hypoxia from the two tissue types.

## 5. Genes Co-Regulated with CSPG4 in SCCs

### 5.1. CSPG4 Co-Expressed Genes in HNSCC and cSCC

A review of the literature suggests a role for CSPG4 in regulating cancer by driving changes in gene expression towards a malignant pathology. To understand how these changes may apply to SCCs, we analysed a list of CSPG4 co-expressed genes available from transcriptome analysis [43,47,145]. The top hundred correlated genes (by Pearson correlation, *R* > 0.46, α = 0.05) were then categorised according to designated function (Supplementary Table S1). Of the top 100 genes 20% had functions associated with cell signalling, 18% with growth and proliferation, 14% with transcription and gene regulation, 11% invasion migration and metastasis, 11% adherence, with the remainder playing roles in immunity, the EMT, and other diverse functions [135]. Notably, many co-regulated genes expressed proteins with roles in regulating cell-ECM interactions, or are themselves ECM components, such as PDPN [160], FLRT2 [161], LAMC2 [162], ITGA3 [163], TENM3 [164], and COL17A [165]. Another group play key roles in cellular differentiation, such as SNAI2 [166], and WNT7A [167] (Supplementary Table S1).

Of the top ten genes by correlation in HNSCC [47], many were found to have functions that support the known role that CSPG4 plays in cancer pathogenesis, such as cell migration, tissue remodelling and angiogenesis. PDPN encodes a mucin-type TM glycoprotein that has functions in development, immunology, migration, invasion, and metastasis [160,168]. Similarly, PIK3CD, which encodes the delta subunit of PI3K, is a key intracellular mediator of cell growth, proliferation, survival and the EMT [169]. Notably, LAMC2, which encodes the γ subunit of laminins, has been linked to severe junctional EB (JEB, formerly Herlitz JEB), providing a further link between EB patients and the development of aggressive SCCs [162,170]. Another link is PLEC, which encodes an intermediate filament binding protein, the lack of which causes EB simplex with muscular dystrophy [171]. Other genes strongly correlated with CSPG4 included: (i) ITGA3, which like all integrins plays important roles in cell surface adhesion [163]; (ii) FLRT2, which encodes a TM cell adhesion protein that regulates embryonic vasculogenesis and neural development, and which has been linked to colorectal cancer [161,172,173]; (iii) CYP26B1, which is a key regulator of all-trans retinoic acid levels and has been implicated in T cell differentiation [174]; (iv) CAVIN1, which codes for an essential protein in the formation of caveolae [175]; (v) MN1, which is an oncogene linked to meningioma and is also associated with developmental defects [176]; and (vi) APP, which is a cell surface receptor and TM precursor protein that is cleaved by secretases to form multiple smaller peptides, which have been found to play a diverse variety of roles in transcription, as antimicrobial peptides and in the formation of amyloid plaques found in Alzheimer’s disease [177].

### 5.2. Comparison with Results of Single Cell Analysis

The result of gene expression correlation analysis in tissues showed significant agreement with transcriptome single cell analysis, recently conducted in cSCC and HNSCC tumour cells [178,179]. Both LAMC2 and ITGA3 mark the partial EMT (or p-EMT) group as identified by Puram et al. (2017), which also contains some of the genes associated with the ECM and has features linked to the canonical EMT [178]. Interestingly, while the p-EMT group lacked expression of most of the classic EMT transcription factors, it does include SNAI2 (expressed by 70% of HNSCC cells). The growth factor, TGFβI, which is also coregulated with CSPG4 in HNSCC, was also the ‘top scorer’ in the p-EMT program [178]. Taken together this puts CSPG4 squarely within the p-EMT subtype, which is unsurprising as these p-EMT cells are localised to the leading edge of a tumour and are associated with increased invasiveness and motility, both of which are closely linked to CSPG4 function [178]. In a more recent study, Ji et al. (2020) [179] further subdivided tumour and non-tumour keratinocytes into four subsets, three of which approximated normal epidermal states (basal, cycling & differentiating), and one being unique to malignancy, referred to as the tumour-specific keratinocyte (TSK) subset. Genes marking the TSK subtype are linked to movement and ECM disassembly, which are both key pathological functions linked to CSPG4 activity. Notably, the TSK signature phenocopies the p-EMT group identified by Puram et al. (2017) [178] demonstrating remarkably similar traits of invasion and motility. Other overlaps between the TSK markers and the top CSPG4 co-expressed genes, were also identified including LAMC2, INHBA, and NT5E. Although CSPG4 was not identified as part of either subset in single cell transcriptome analysis, the finding that CSPG4-associated genes form part of both the p-EMT and TSK signatures further illuminate the role of CSPG4 in SCC pathogenesis.

### 5.3. CSPG4 Co-Expressed Genes in RDEB SCCs

**Figure 6:**
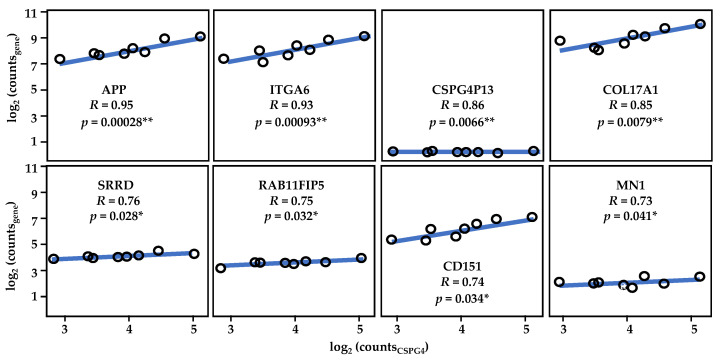
Results of re-analysis of RDEB-SCCs transcriptome data. Shown, the strong correlation between CSPG4 and ITGA6, as well as that of CSPG4 and COL17A1. CSPG413 is a pseudogene which is co-expressed with CSPG4 (by Pearson Correlation, * *p* < 0.05, ** *p* < 0.01; α = 0.05). Figure derived from re-analysis of publicly available published data: GSE130925 [43] and GSE130925 [145]. See also Supplementary Table S1.

To understand the function of CSPG4 through its relationship with other expression programs, RDEB-SCC transcriptome re-analysis was also performed. Several genes were found to be significantly co-expressed with CSPG4 in RDEB-SCCs. When this list was compared with the larger HNSCC data set (from the TCGA), 25 genes were found to significantly correlate with CSPG4 in both HNSCC and RDEB-SCCs (*p* < 0.05, *R* > 0.46, for both HNSCC & RDEB-SCC, by Pearson correlation, α = 0.05) (Supplementary Table S1, Figure 6). In addition to APP and MN1 (see above), this list also included but was not restricted to: (i) the pseudogene CSPG4P13; (ii) ITGA6 and COL17A1, which are both linked to subtypes of JEB [180,181,182]; (iii) SRRD, which is linked to cell proliferation [183]; (iv) RAB11FIP5, which is linked to cell polarisation [184]; and (v) the surface glycoprotein implicated in keratinocyte migration, CD151 (aka 4TM) [185]. The fact that 75 genes showed lower correlation or inverse correlation (Supplementary Table S1) is unsurprising given the heterogeneity of HNSCC subtypes. However, the common link with COL17A and ITGA6 is interesting, due to the synergistic role that integrins, collagen XVII and CSPG4 play in skin stem cell function [139,140,141].

### 5.4. Skin Stem Cells, the p-EMT/TSK Programs and CSPG4

As mentioned above, CSPG4 expression seems to be linked to a particular group of skin stem cells, and expression corelates with self-renewal in situ, as well as expression of stem cell factors, such as integrin (e.g., ITGA6), and laminins (esp. Lam332). The boundary between the epidermis and the dermis is established by a basement membrane, onto which keratinocytes of the basal layer are anchored by integrin receptors in junctional complexes (hemidesmosomes) [186,187,188]. During cell migration and wound healing, β1 integrins provide TM connections to the actin network through recruitment of effectors to their CTDs [186]. From one side, integrin α6β4 is tethered to intermediate filaments by plectin and bullous pemphigoid antigens (e.g., BP180). At the other end it binds to laminin 332, linking keratins to type VII collagen dermal anchoring fibrils. By transmitting signals to effect changes in gene expression [186], these junctionally localised cytoskeletal proteins control the maintenance of stem cell populations in the epidermis.

Recently it has been reported that the expression of the hemidesmosome component collagen XVII by epidermal stem cells fluctuates physiologically through genomic/oxidative stress-induced proteolysis. In addition, clones that express high levels of COL17A1, divide symmetrically, out compete, and eliminate adjacent clones that express lower levels of COL17A1, and divide asymmetrically. Stem cells with higher potential are thus selected for homeostasis. The eventual loss of COL17A1 limits their competitive ability, thereby causing ageing [189]. Recent single cell gene expression analysis has confirmed that COL17A marks the ‘basal’ cell subpopulation, which was one of three keratinocyte groups identified in normal skin: the others being ‘cycling’, and ‘differentiating’ cells, marked by MKI67 and KRT1, respectively. In addition, to also marking a basal tumour cell population, COL17A was also found to mark TSKs (see above) [179]. Given that competition is a key feature of normal skin homeostasis, mediated by COL17A1, it is interesting to speculate whether cancers acquire certain features of normal skin stem cells, such as expression of COL17A/ITGA6/CSPG4, to facilitate self-renewal, and outcompete non-cancerous cells in healthy skin.

## 6. CSPG4 as a Diagnostic Marker in Cancer

The limited number of clinically useful guides that can be used in SCC diagnosis and treatment has driven research into molecular markers that might provide an objective determination of clinical course [42,43,145]. This work has been particularly critical for those patients for which early diagnosis is key to treatment success, or for which no (or few) clinical treatments are available, including the aged, immunosuppressed and those with rare genetic disorders such as RDEB [31,32,33,34,35,36,37]. Recent transcriptome analysis has revealed several useful candidates that might serve this purpose [43,47,49,145]. The focus of this review was on the cell surface marker CSPG4. The extensive link to melanoma and the array of tools available to study it mean that we understand a lot more about the structure and function of CSPG4 than other potential candidates. Published research has also shown a functional similarity between the role of CSPG4 as a mediator of cell differentiation, motility and tissue turnover in development, and its role in mediating key aspects of malignancy in cancer (Table 1 and Table 2). However, much of the function of CSPG4 in pathophysiology remains relatively undefined. To understand a possible role for CSPG4 as a clinical marker we first examined the relationship of CSPG4 expression to malignancy.

The first role of a tumour biomarker is to distinguish tumour cells from the non-tumour tissues from which they arise [129]. What is clear from transcriptome analysis and primary research at the bench, is that increased CSPG4 expression is a feature of SCCs, regardless of origin [43,47,57,134,145] (Figure 4 and Figure 5). However, in our reanalysis of recently published transcriptome data, we have demonstrated that the tumour microenvironment is a key consideration in these studies, with the degree of difference in CSPG4 expression reduced when cells are examined in culture (Figure 4b). We have also shown that while CSPG4 expression in melanoma might be linked to pro-survival, a drop in expression in HNSCCs linked to the change in a tumour from a pre-metastatic to a metastatic clinical stage was also observed (Figure 5). A finding that is supported by recent work from the Ranson Laboratory [42], showing higher CSPG4 expression in cSCCs that had not yet metastasized. Interestingly, in several non-SCC cancer studies, CSPG4 expression has been linked to characteristics associated with reduced malignancy. In a sarcoma study, CSPG4 deletion at the time of tumour initiation, and before establishment of the tumour, resulted in larger tumours, while suppression in established tumours resulted in reduced tumour growth [85]. Another, study, showed that although expression of CSPG4 was higher in non-malignant naevi, a lower level of expression of CSPG4 in conjunctival melanoma was associated with higher risk of recurrence [190]. While these results do not discount the use of CSPG4 as a diagnostic marker, they do paint a more complex and nuanced picture of its potential application; where tumour cell CSPG4 expression is dependent on stage, as well as the changing tumour microenvironment.

**Figure 7:**
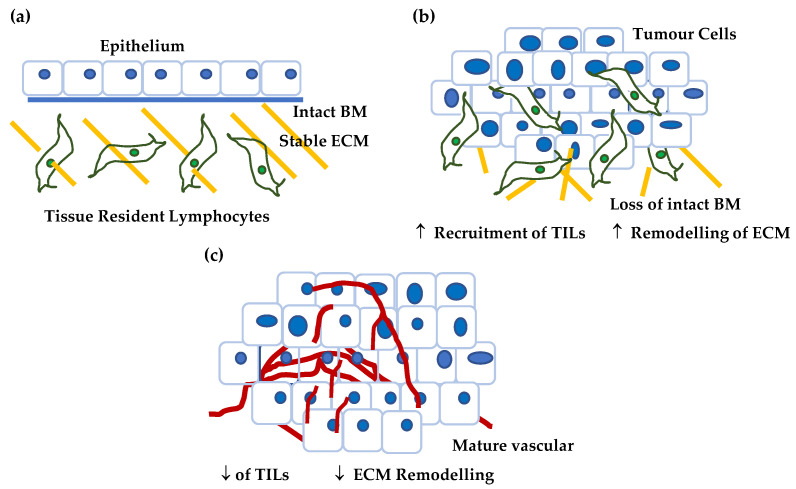
Proposed model for CSPG4 expression in cancer. (**a**) In normal, non-tumour tissue (in this case skin) the tissue is characterised by differentiated epithelium, low levels of tissue remodelling, a stable ECM, and quiescent tissue resident lymphocytes. Normal skin expresses little if any CSPG4. (**b**) As a tumour develops and grows the tissue is characterised by: (i) epithelium that becomes undifferentiated, as basement membrane barriers are broken down, (ii) ECM remodelling associated by tumour invasion, and (iii) increased hypoxia, a condition that is associated with the recruitment of tumour infiltrating lymphocytes (TILs). In addition, the number of tumour stem cells increases with the acquisition of markers of self-renewal and invasion (CSPG4, ITGA6, e.g., COL17A1 etc). This premetastatic lesion is characterised by higher CSPG4. (**c**) As the tumour develops further it acquires an established vascular network, and a stable tumour microenvironment where hypoxia is reduced and there is a reduction in TILs. There is a reduction in the number of cells capable of self-renewal as a proportion of the tumour mass. At this later stage CSPG4 levels are lower.

In addition, to transcriptome analysis, we conducted functional analysis on CSPG4, as well as examined genes coregulated with CSPG4 in SCCs. Analysis of CSPG4 structure and function shows that it plays a key role in tissue turnover and regulation of the ECM, either directly or indirectly. Unsurprisingly, genes that correlated with CSPG4 in SCCs (*p* < 0.05, *R* > 0.46, by Pearson correlation) had similar functions in growth and proliferation, adhesion as well as invasion, migration, and motility. Notably, COL17A, LAMC2 and ITGA3, all of which have been linked to aggressive SCCs, are highly correlated in HNSCCs and RDEB-SCCs and play important roles in epidermal stem cell and self-renewal (Figure 6). The fact that these genes are co-regulated with CSPG4 may suggest that SCCs acquire a stem cell phenotype as part of the pathology of malignancy, which is closely aligned to that shown by non-cancer skin stem cells [186,187,188,189]. Whether this reflects the tumour cell’s epidermal ancestors, or a phenotype selected to mimic the behaviour of these same stem cells remains unknown. Taken together this information leads to a dynamic model of CSPG4 expression in malignancy, one that is dependent on stage and the microenvironment (Figure 7).

CSPG4 expression is clearly linked to malignancy in tumour cells, however, what this model shows is that understanding how dynamic changes in CSPG4 expression can be used for clinical diagnosis will be challenging. One way to overcome this difficulty is by dynamically tracking changes in CSPG4 expression through analysis of the liquid biopsy. Previous studies have found elevated levels of soluble CSPG4 in melanoma patient serum, but there remains disagreement about the relationship between CSPG4 level in the blood and patient prognosis [132,133]. Notably, studies using CSPG4 as a marker for circulating tumour cells, which may provide cellular context, have yet to be conducted, and future work in this area will shed further light on the utility of CSPG4 as a diagnostic blood marker. In addition, there have been surprisingly few studies which have examined CSPG4 protein expression in SCCs. One of these was from 1981 and only used indirect immunofluorescence with UV [57]. More work using multiple sophisticated techniques, such as FACS analysis and confocal microscopy need to be performed, on a larger range of SCCs from different sources, to properly elucidate the utility of CSPG4 as diagnostic marker.

## 7. Conclusions

Using a combination of literature searching and reanalysis of published data this review has sought to illuminate the dynamic and multivariate roles that CSPG4 might play in cancer progression, and to assess CSPG4 expression as a diagnostic marker in aggressive SCCs. This is particularly important given that CSPG4 targeting has been suggested as an anti-cancer treatment, however, therapies have shown minimal efficacy in patients. It might be that treatment needs to develop in conjunction with a companion diagnostic that considers the complex and dynamic nature of CSPG4 expression. Reanalysis of published data has also revealed a surprising link between CSPG4 and well known TSK and p-EMT gene clusters. A relationship that has not previously been identified. Taken as a whole, what this work has also demonstrated is that assessment of CSPG4 as a tumour marker or therapeutic target in SCC should not be conducted independently of stage, and the tumour environment.

## Figures and Tables

**Table 1:** CSPG4 expression in non-cancer tissues.

Author/Source	Cell/Tissue/Organ Type			Protein Levels	Median Expression **	Type (A/E) *
The Human Protein Atlas and Uhlén et al. [49] Huret et al. [50]	Brain	Cerebral cortex		High		A
		Cerebellum		High		
		Hippocampus		High		
		Caudate		Medium		
	Endocrine	Thyroid		Medium		A
		Parathyroid		Medium		
		Adrenal gland		Medium		
	Respiratory	Nasopharynx		High		A
		Bronchus		High		
		Lung		Medium		
	Proximal digestive tract	Oral mucosa		Low		A
		Salivary gland		Medium		
		Esophagus		Medium		
	Gastrointestinal tract	Stomach		Medium		A
		Duodenum		High		
		Small intestine		High		
		Colon		Medium		
		Rectum		Medium		
	Liver			Medium		A
	Gallbladder			High		A
	pancreas			High		A
	Kidney			Medium		A
	Bladder			Medium		A
	Male reproductive organs			Medium		A
	Female reproductive organs	Vagina		Medium		
		Ovary		Medium		
		Fallopian tube		Medium		
		Endometrium		High		
		Cervix		Medium		
		Placenta		Medium		
	Breast			Medium		
	Muscle	Heart muscle		Medium		A
		Smooth		Low		
		Skeletal		Medium		
	Adipose tissue and soft tissue			Low-Medium		A
	Skin			Medium		A
	Lymphoid tissue	Bone marrow		Medium		A
		Appendix		High		
		Spleen		Medium		
		Lymph node		Medium		
		Tonsil		Medium		
	Haematopoietic			Low		A
*** GTEx data, Carithers et al. [51]	Lung				37.81	A
	Vascular	Aorta			175.6	
		Coronary artery			132.6	
		Tibial artery			246.2	
	Heart	Atrial appendage			12.85	
		Left ventricle			8.040	
	Brain	Amygdala			10.48	
		Anterior cingulate cortex			8.864	
		Caudate			6.361	
		Cerebellar hemisphere			3.078	
		Cerebellum			3.845	
		Cortex			7.710	
		Frontal cortex			6.361	
		Hippocampus			8.213	
		Hypothalamus			8.861	
		Nucleus accumbens			6.160	
		Putamen			5.692	
		Spinal cord			5.574	
		Substantia nigra			8.926	
	Nerve (tibial)				66.59	
	Pituitary				2.565	
	Thyroid				11.30	
	Liver				0.5535	
	Pancreas				1.326	
	Spleen				9.568	
	Stomach				10.87	
	Small intestine				10.92	
	Colon	Sigmoid			105.7	
		Transverse			35.10	
	Esophagus	Gastroesophageal junction			98.16	
		Mucosa			7.547	
		Muscularis			98.31	
	Cervix	Ectocervix			29.65	
		Endocervix			28.90	
	Female reproductive organs	Fallopian tube			37.31	
		Ovary			12.14	
		Uterus			60.30	
		Vagina			19.53	
	Breast				23.63	
	Male reproductive organs		Prostate		23.09	
			Testis		3.153	
	Kidney		Medulla		6.372	
			Cortex		4.208	
	Adrenal gland				2.933	
	Bladder				59.06	
	Adipose		Subcutaneous		51.61	
			Visceral		33.41	
	Skeletal muscle				23.65	
	Fibroblasts (cultured)				14.35	
	Skin		Sun exposed		13.22	
			Non sun exposed		10.93	
	Whole blood				0.09142	
Nishiyama et al. [136]	Chondroblast precursors					E
Fukushi et al. [48]	Chondroblast precursors					E
	Osteoblast precursors					
Ozerdem et al. [54], Grako et al. [137]	Cardiomyocytes					E
	Pericytes					E & A
	Vascular smooth muscle					E
Midwood et al. [138]	Chondrocytes					A
Ghali et al. [139]	Epidermal and interfollicular progenitor cells					A
Legg et al. [140], Giangreco et al. [141]	Interfollicular epidermis progenitor cells					A
Schiffer et al. [123]	Oligodendrocyte precursor cells					A
Trotter et al. [142]	Oligodendrocyte precursor cells					A
	Protoplasmic astrocytes					
	Neurons					
Kozanoglu et al. [143]	Bone marrow mesenchymal cells					A

* A—Adult, E—Embryonic; ** TPM—Transcripts per million, *** GTEx—Genotype-Tissue Expression.

**Table 2:** CSPG4 expression in malignancy.

Author/Source	Tumour Type		% CSPG4^+^ Lesions Compared to Total	Rel. Expression Compared to Normal
Wilson et al. [57]	Melanoma		98.3–100%	
	NMSC	cSCC	37.5–50%	
		Basal cell carcinoma		
Nishi et al. [146]	Melanoma	Acral lentigous melanoma (ALM)	53.6%	Increased; staining intensity for ALM was weaker than SSM
		Superficial spreading melanoma (SSM)	100%	
Kageshita et al. [147]	Melanoma	Primary	50%	
		Metastatic	83.3%	
Beard et al. [148]	Melanoma			Increased
	Triple Negative breast Cancer (TNBC)			Increased
	Glioblastoma			Increased
Tang et al. [47]	Pancreatic adenocarcinoma			Increased
	Pheochromocytoma and paraganglioma			
	Esophageal carcinoma			
	HNSCC			
	Brain lower grade glioma			
	Glioblastoma multiforme			
	Kidney renal clear cell carcinoma			
	Melanoma			
Uhlen et al. [49]	Renal cancer			
	Urothelial cancer			
	Glioma			
Warta et al. [134]	HNSCC			Increased
Schwab et al. [149]	Chordoma and chondrosarcoma			Present
Wang et al. [150]	Breast cancer	ER−/PR−/HER2−(TNBC)	72.7%	
		ER^+^	28.6%	
		HER2+	16.7%	
Behm et al. [131]	Acute lymphoblastic leukemia		8.6%	
Petrovici et al. [130]	Acute myeloid leukemia		50%	
Rivera et al. [151]	Malignant mesothelioma		60.98%	
Keleg et al. [129]	Pancreatic carcinoma	Adenosquamous carcinoma		Increased
		Anaplastic ductal adenocarcinoma		
		Intraductal papillary mucinous neoplasm with associated invasive carcinoma		
Riccardo et al. [152]	Osteosarcoma			Increased

## Data Availability

Figures presented in this work (notably Figure 3, Figure 4, Figure 5 and Figure 6), were constructed from re-analysed published and publicly available data from the TCGA data base, accessed through the GEPIA website (http://gepia.cancer-pku.cn, accessed 9 September 2022) [47], and transcriptome data for RDEB-SCCs, available through the NCBI, Gene Expression Omnibus (GEO) (https://www.ncbi.nlm.nih.gov/geo, accessed 9 September 2022); accession numbers, GSE130925 [43] and GSE130925 [145].

## Supplementary Materials

The following supporting information can be downloaded at: https://www.mdpi.com/article/10.3390/cancers14225564/s1, Table S1: Top 100 genes correlated with CSPG4 in HNSCC.
