# Supplementary material for: Chondroitin Sulfate Proteoglycan 4 as a Marker for Aggressive Squamous Cell Carcinoma
Source: Cancers (Basel). 2022 Nov 13;14(22):5564. doi: 10.3390/cancers14225564 (PMC9688099; doi:10.3390/cancers14225564)
Supplement: Supplementary file 1 [file cancers-14-05564-s001.zip › cancers-1975952-supplementary.pdf]

Review

Chondroitin sulfate proteoglycan 4 as a marker for aggressive squamous cell carcinoma

Kathryn Chen [1,2], Joel Yong [1,3], Roland Zauner [4], Verena Wally [4], John Whitelock [1,5], Mila Sajinovic [1], Zlatko Ko-pecki [6], Kang Liang [1,3], Kieran Francis Scott [1,2] and Albert Sleiman Mellick [1,5,*]

Supplementary Data

Supplementary Table S1. Top 100 genes expressed in HNSCC and correlated with CSPG4 ($R_1 > 0.46$, by Pearson correlation). Data derived from TCGA [47]. Also shown correlation of CSPG4 with genes in RDEB-SCCs (R_2) [43,145]. Genes significantly correlated with CSPG4 in both HNSCC, and RDEB-SCC are indicated #, and bolded (R > 0.46, both HNSC & RDEB-SCC). Also shown gene function based on OMIM ontology analysis [135]. ADH – adhesion, EMT – epithelial to mesenchymal transition, GPS – growth, proliferation, survival, IMM – invasion, migration, metastasis, IMU – immune, OTH – other; SIG – signalling, TRA – miscellaneous cell transport events, TRN – transcription.

	Ensembl	R1	R2	OMIM
PDPN	ENSG00000162493.16	0.61	0.32	IMM, GPS, ADH
PLEC	ENSG00000178209.14	0.61	-0.48	ADH
FLRT2	ENSG00000185070.10	0.6	-0.07	ADH
CYP26B1	ENSG00000003137.8	0.59	-0.52	IMU
**MN1**	**ENSG00000169184.5**	**0.58**	**0.73#**	**TRN**
PIK3CD	ENSG00000171608.15	0.58	-0.67	GPS, SIG, EMT
PTRF	ENSG00000177469.12	0.58	-	TRA, SIG
**LAMC2**	**ENSG00000058085.14**	**0.57**	**0.95#**	**ADH, IMM, SIG**
**APP**	**ENSG00000142192.20**	**0.57**	**0.49#**	**TRN**
ITGA3	ENSG00000005884.17	0.57	0.19	ADH
**SIRPA**	**ENSG00000198053.11**	**0.56**	**0.85#**	**SIG**
**SNAI2**	**ENSG00000019549.8**	**0.56**	**0.61#**	**TRN, EMT**
**COL17A1**	**ENSG00000065618.16**	**0.56**	**0.54#**	**ADH**
PMEPA1	ENSG00000124225.15	0.56	0.42	SIG
LAMA3	ENSG00000053747.15	0.56	0.25	ADH, IMM, SIG
LRRC8C	ENSG00000171488.14	0.56	-0.1	TRA
**TENM3**	**ENSG00000218336.7**	**0.55**	**0.86#**	**OTH**
**ACTN1**	**ENSG00000072110.13**	**0.55**	**0.6#**	**ADH, IMM**
LTBP1	ENSG00000049323.15	0.55	-0.09	SIG
CSMD2	ENSG00000121904.17	0.55	-0.19	IMM
WNT7A	ENSG00000154764.5	0.55	-0.37	SIG
CSPG4P13	ENSG00000260139.6	0.55	-0.81	Other
APBB2	ENSG00000163697.16	0.54	0.11	TRN
SDK2	ENSG00000069188.16	0.54	-0.22	ADH
ANTXR2	ENSG00000163297.16	0.54	-0.59	ADH
**PRNP**	**ENSG00000171867.16**	**0.53**	**0.68#**	**NA**
**SLC12A4**	**ENSG00000124067.16**	**0.53**	**0.57#**	**NA**
KIAA1644	ENSG00000138944.7	0.53	0.42	Other
ANXA5	ENSG00000164111.14	0.53	0.33	GPS, TRA, SIG
PXN	ENSG00000089159.15	0.53	0.32	ADH
SPON2	ENSG00000159674.11	0.53	0.11	ADH
STON2	ENSG00000140022.9	0.53	0.05	TRA

IRS1	ENSG00000169047.5	0.53	-0.12	TRA
CD276	ENSG00000103855.17	0.53	-0.33	IMU
TNFRSF12A	ENSG00000006327.13	0.53	-0.49	SIG
LAMB3	ENSG00000196878.12	0.53	-0.72	GPS
RTN4	ENSG00000115310.17	0.53	-	GPS
**ITGA5**	**ENSG00000161638.10**	**0.52**	**0.93**[#]	**SIG**
**ITGA6**	**ENSG00000091409.14**	**0.52**	**0.56**[#]	**IMM**
VEGFC	ENSG00000150630.3	0.52	0.44	GPS, TRN
SLC39A13	ENSG00000165915.13	0.52	0.13	TRA
CYP27C1	ENSG00000186684.12	0.52	0.08	TRN
MUL1	ENSG00000090432.6	0.52	-0.63	SIG
AJAP1	ENSG00000196581.10	0.51	0.36	IMM
AC108142.1	ENSG00000177822.7	0.51	0.26	Other
INHBA	ENSG00000122641.9	0.51	0.11	GPS, EMT
EFNB1	ENSG00000090776.5	0.51	-0.35	GPS, TRN
PLCD4	ENSG00000115556.13	0.51	-0.67	SIG
EXT1	ENSG00000182197.10	0.51	-0.71	GPS
GNA12	ENSG00000146535.13	0.51	-	GPS, IMM. SIG
**EDNRA**	**ENSG00000151617.15**	**0.5**	**0.74**[#]	**TRN**
**PLEK2**	**ENSG00000100558.8**	**0.5**	**0.65**[#]	**GPS, IMM**
**ITGB1**	**ENSG00000150093.18**	**0.5**	**0.53**[#]	**GPS**
EHD2	ENSG00000024422.11	0.5	0.43	TRA
GRIA3	ENSG00000125675.17	0.5	-0.21	TRA
RP11-497E19.1	ENSG00000205562.2	0.5	-0.32	Other
SERPINE1	ENSG00000106366.8	0.5	-0.34	TRN
KIRREL	ENSG00000183853.17	0.5	-0.35	Other
CD151	ENSG00000177697.17	0.5	-0.41	GPS, SIG
COL4A2	ENSG00000134871.17	0.5	-0.64	GPS
SEMA3C	ENSG00000075223.13	0.5	-	GPS
CLMP	ENSG00000166250.11	0.5	-	ADH
**PLAU**	**ENSG00000122861.15**	**0.49**	**0.75**[#]	**IMM**
**P4HA2**	**ENSG00000072682.18**	**0.49**	**0.66**[#]	**GPS, SIG**
**VAV2**	**ENSG00000160293.16**	**0.49**	**0.66**[#]	**SIG**
**ACTR1A**	**ENSG00000138107.11**	**0.49**	**0.66**[#]	**TRA**
**RAB11FIP5**	**ENSG00000135631.15**	**0.49**	**0.64**[#]	**TRA**
**RP11-167H9.3**	**ENSG00000243321.3**	**0.49**	**0.63**[#]	**Other**
**LUZP1**	**ENSG00000169641.13**	**0.49**	**0.59**[#]	**SIG**
ESYT1	ENSG00000139641.12	0.49	0.31	GPS, TRA
PDGFC	ENSG00000145431.10	0.49	0.17	GPS
DSE	ENSG00000111817.16	0.49	-0.09	IMU, TRN
PANX1	ENSG00000110218.8	0.49	-0.11	SIG
DKK3	ENSG00000050165.17	0.49	-0.12	SIG
TLL1	ENSG00000038295.7	0.49	-	Other
**TSPAN5**	**ENSG00000168785.7**	**0.48**	**0.76**[#]	**GPS, ADH, IMM**
**SRRD**	**ENSG00000100104.12**	**0.48**	**0.62**[#]	**Other**
FBLIM1	ENSG00000162458.12	0.48	0.16	ADH
MSN	ENSG00000147065.16	0.48	0.01	IMM, SIG
SLC9A1	ENSG00000090020.10	0.48	-0.57	Other
CHSY1	ENSG00000131873.5	0.48	-0.6	GPS
COL16A1	ENSG00000084636.17	0.48	-0.61	Other
EHD1	ENSG00000110047.17	0.47	0.62	TRA
PTPN21	ENSG00000070778.12	0.47	0.31	GPS, SIG
LIMA1	ENSG00000050405.13	0.47	0.24	ADH

PLOD1	ENSG00000083444.16	0.47	-0.3	TRN
CLSTN1	ENSG00000171603.16	0.47	-0.5	TRA, SIG
TSHZ3	ENSG00000121297.6	0.47	-0.74	TRN, SIG
**SORCS2**	**ENSG00000184985.16**	**0.46**	**0.49#**	**SIG**
PHLDB1	ENSG00000019144.16	0.46	0.39	GPS, EMT
VDR	ENSG00000111424.10	0.46	0.19	TRN
PTK7	ENSG00000112655.15	0.46	0.14	IMM
COL4A1	ENSG00000187498.14	0.46	0.13	TRN
AFAP1L1	ENSG00000157510.13	0.46	0.05	GPS
TGFBR2	ENSG00000163513.17	0.46	-0.12	TRN, SIG
TGFBI	ENSG00000120708.16	0.46	-0.18	TRN
SCRN1	ENSG00000136193.16	0.46	-0.44	Other
MYH9	ENSG00000100345.20	0.46	-0.48	TRN
MMP14	ENSG00000157227.12	0.46	-0.58	TRN
NT5E	ENSG00000135318.11	0.46	-0.58	TRN
